# Application of Waste Glass Powder for Sustainable Concrete: Design, Performance, Perspective

**DOI:** 10.3390/ma18040734

**Published:** 2025-02-07

**Authors:** Changshun Zhou, Mingyong Li, Quang Dieu Nguyen, Xuqun Lin, Arnaud Castel, Yu Pang, Zhizhong Deng, Tianxing Shi, Chao Mai

**Affiliations:** 1 Yuanpei College, Shaoxing University, Shaoxing 312000, China; changshun0121@163.com (C.Z.); mingyongliyp@163.com (M.L.); 2 School of Civil and Environmental Engineering, University of Technology Sydney, Ultimo, NSW 2007, Australia; arnaud.castel@uts.edu.au (A.C.); yu.pang@student.uts.edu.au (Y.P.); zhizhong.deng@student.uts.edu.au (Z.D.); tianxing.shi-1@student.uts.edu.au (T.S.); chao.mai@student.uts.edu.au (C.M.)

**Keywords:** waste glass powder, mechanical properties, durability, sustainability, review

## Abstract

Waste glass (WG) is a solid waste with increasing reserves, and its disposal has become a global issue. The application of WG in the construction industry is one of the promising pathways for recycling WG while reducing high-cost WG landfills. This study is conducted to systematically review the potential effects of WG as both a cement and aggregate replacement on the mechanical and durability properties of cementitious composites. Different waste glass powders (WGP) can lead to various effects on both the workability and hydration of cementitious composites. 10 to 20 wt% WGP as supplementary cementitious materials (SCMs) is the optimal dosage to promote the mechanical and durability properties of the WGP–cement composites. The presence of WGP can promote the chemical resistance, freeze–thaw resistance, and elevated temperature resistance of WGP–cement composites. However, more studies and experiments are needed to provide conservative conclusions on how WGP would affect the durability properties of both normal and high-strength concrete. Sustainable concrete technology requires the use of low-carbon materials while maintaining long-term structural resilience. There is an increasing trend in recycling WGP as a cement or aggregate replacement in designing green concrete.

## 1. Introduction

Global rapid civilisation progress and continuous development of living standards poses a raising demand in glass across different sectors. For example, waste glass (WG) accounts for approximately 7% of the total solid waste in China [1]. Furthermore, the global annual amount of WG landfills is approximately 200 million tons [2,3]. Thus, proper treatment of WG has been an important environmental issue around the globe.

Recycling WG in the construction sector could not only alleviate the demand in land resources and economic burden due to WG landfills but also promote a sustainable resource utilisation and low-carbon development of building designs. Since Schmidt and Saia first [4] used WG as building materials, extensive studies have been conducted to investigate WG as a favourable alternative to replace cement or fine aggregates. Due to the diversity in types, particle sizes, and chemical compositions of WG, different effectiveness and performances are observed. Borhan [5] ground waste glass into waste glass powder (WGP) to partially replace fine aggregates. He found that the mechanical properties of concrete with WGP decreased when the dosage of WGP increased, having a good agreement with Yu et al. [6]. However, several studies [7,8] reported that the addition of WGP could improve the mechanical properties of concrete when compared to the reference group. Replacing cement with 30% WGP, Serelis et al. [9] reported that a similar compressive strength was achieved for lightweight-aggregate concrete with and without WGP. Madanoust and Ghavell [10] observed that the potential effects of WGP on the mechanical properties of cementitious composites were correlated with the curing age. Although the addition of WGP reduced the early-age mechanical properties, the pozzolanic reactivity of WGP improved the mechanical properties in the long term. Lam et al. [11] added 3 wt% ultra-fine WGP (<0.7 µm) to a cementitious composite and found that the compressive strength of the WGP–cement composites was increased by 10%.

Meanwhile, when WG is used as supplementary cementitious materials (SCM), the workability and durability of WGP–cement composites were improved [12]. Several studies [13,14] reported that WGP with irregular shape reduced the workability of the cementitious composites. Islam et al. [15] 25 wt% WGP replacing cement led to a light increase (up to 3% increase) in the flow. Baikerikar et al. [16] conducted a rapid chloride penetration test (RCPT) on concrete with WGP replacing both cement and sand. They observed a 13.39% reduction in charge passing in samples with 15% cement replacement with WGP and 30% fine aggregate replacement with WG when compared to the control group. More noteworthy, many studies [17,18] suggested that when the particle size of WGP was less than 20 µm, the alkali-silica reactions (ASR) of WGP–cement composites were reduced. Similarly, Shayan and Xu [19] reported that WGP addition promoted satisfactory performance against shrinkage and ASR. The primary reason is that WGP leads to a denser microstructure and better bonding in the interfacial transition zone (ITZ) [20]. Schwarz et al. [21] mentioned that the optimal addition of WGP was approximately 10%, having favourable durability properties.

Since it could be widely used in concrete structures, it is crucial to understand the engineering characteristics of WGP–cement composites. This study aimed to summarise existing studies to examine the physiochemical properties of WGP and analyse the potential effects of WGP on both the mechanical and durability properties of concrete. Finally, the shortcomings and challenges in current applications of WGP in concrete are discussed. As a result, this study could provide valuable data for researchers involved in WGP-related studies, improving the understanding of potential effects of WGP on the mechanical and durability properties of sustainable concrete.

## 2. Physiochemical Properties

**Figure 1:**
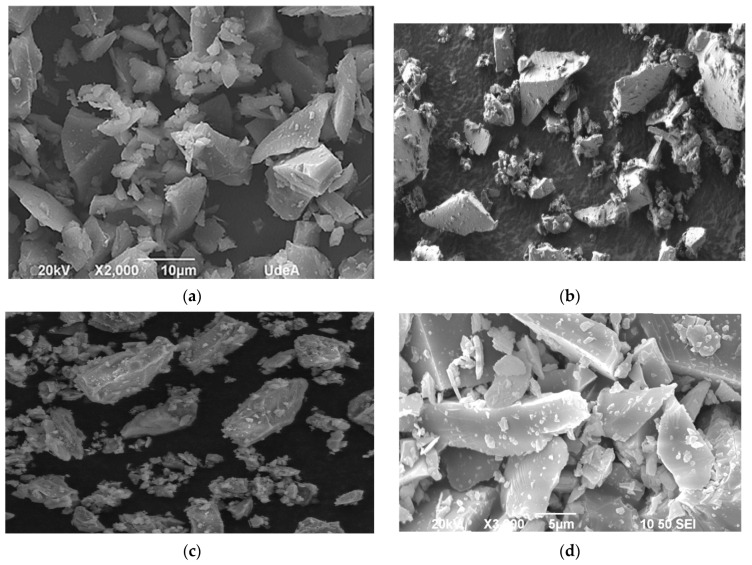
SEM images of different waste glass powders. (**a**) Waste flat glass [23]; (**b**) Waste brown glass [22]; (**c**) Waste green glass [24]; (**d**) Waste transparent glass [13].

Figure 1 shows scanned electron microscopy (SEM) images of different types of glass powder. It could be found that although different types of WGP are investigated [13,22,23,24], similar characteristics are observed, including smooth surfaces and sharp edges. Lu et al. [13] mentioned that due to the sharp edges in WGP, it promoted a better interlocking with the cementitious matrix, providing higher bonding forces. However, since the grinding process in each study is different, the particle size of WGP varies significantly (Figure 1a–d).

Table 1 depicts the chemical compositions of WG originated from different glass sources. The main oxides are SiO_2_, Na_2_O, and CaO. In particular, the content of SiO_2_ is generally higher than 60% (Table 1), revealing potentially a high pozzolanic reactivity. In other words, WGP may have sufficient amorphous silica. There are only small percentages of K_2_O, MgO, and Al_2_O_3_ in WGP (Table 1). However, it could be noted that the silica content in the study by Muhedin et al. [25] and Ban et al. [26] is 59.7% and 54%, respectively, being lower than those mentioned in other studies. This difference highlights the variety of glass types designed for different commercial purposes. The second highest oxide contents are Na_2_O and CaO, located between 8.7–14.7% and 7.62–11.84%, respectively. However, Ban et al. [26] reported that the CaO was only 0.85%, being significantly different from the content of CaO mentioned in other studies. Pereira et al. [27] reported that the pozzolanic reactivity of WG was highly related to its particle size. Similarly, Mirzahosseini and Riding [28] found that relatively finer WGP (<25 µm) had better pozzolanic reactivity than coarser WGP (25~38 μm and 63~75 μm).

**Figure 2:**
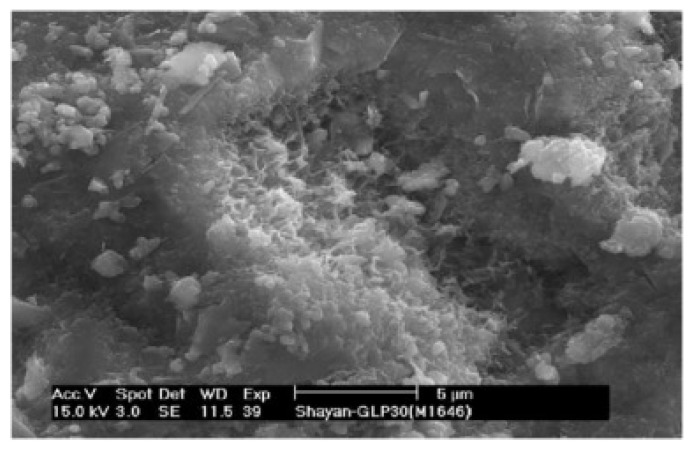
C-S-H gel near WGP [19].

According to several studies [37,38], at an early age, WGP used as SCM reduces the cement hydration due to the dilution effect of cement. However, at a later curing age, the pozzolanic reactions of WGP consume calcium hydroxide generated by cement hydration, forming more C-S-H gels [37,38]. Du et al. [39] reported that complete pozzolanic reactions were achieved when the content of WGP was in the range of 30–45 wt%. As depicted in Figure 2, many needle-like gels were observed on the surface of WGP, indicating that amorphous silica reacted with calcium hydroxide to form additional C-S-H gel [19]. Maraghechi et al. [40] reported that the calcium to silicon ratio (C/S ratio) was in the range of 0.6–1.4 in the presence of WGP, being different from the C/S ratio (1.2–2.3) in the C-S-H gel from cement hydration [41,42].

## 3. Influence on Wet-State Cementitious Composites

### 3.1. Workability

Table 2 summarises the potential effects of WGP used for both cement or fine aggregate replacement on the workability of the cementitious composites, highlighting different effects of WGP addition on the workability. Using WGP as SCMs, Lu et al. [13] reported that 20 wt% WGP to replace cement led to a 29% reduction in the flow, due to the sharp edge of the WGP (0.5 h grinding with size < 800 µm). Similarly, Shayan & Xu [19] observed that, when replacing cement with 30 wt% WGP, up to a 14.3% reduction in flow was found. However, Schwarz et al. [43] reported that an increase in cement replacement by WGP (size < 88 µm) led to an increase in the flow. They mentioned that the flow increase was attributable to the low water adsorption of WGP, leading to more free water in the cementitious mixture.

For fine aggregates replaced by WGP, Lu et al. [46] reported that using WGP to replace sand up to 100 wt%, the flow was increased up to 26.9%. Maglad et al. [52] replaced sand up to 50 wt%, and they observed that the slump increased by 3.22% for the sample with 30 wt% WGP. Maglad et al. [52] revealed that the workability improvement was attributed to the smooth texture and finer particle size of WGP when compared to natural sand. Similarly, Bahadur et al. [53] found that, after replacing river sand by 20 wt%, the workability of the WGP–cement composites increased. They suggested that due to the finer particle size, the addition of WGP increased the slump value of the cementitious composites. Ali & Al-Tersawy [48] found that the slump was increased by 37.5% for samples with 50 wt% WGP replacing river sand. They mentioned that due to the low water absorption ratio, the presence of WGP led to an increase in slump. However, Bisht & Ramana [50] observed that, due to the angular shape of WGP replacing fine aggregates, the workability of WGP–cement composites was reduced. Park et al. [14] found that the workability was reduced up to 44.3% for samples with 70 wt% WGP replacing sand when compared to the control group. Steyn et al. [54] also reported that after replacing sand by 15–30 wt%, the WGP–cement specimens had a reduced slump. They highlighted that, due to the uneven surface texture of WGP, higher flow resistance was observed when compared to WGP-free samples. A similar conclusion was drawn by Tan et al. [55], reporting that the sharper edge of WGP when compared to normal sand led to a reduction in the flowability of the WGP mortar. Several studies [55,56] explained that when using WGP as fine aggregates, the slump of concrete reduced due to the polygonal shape of WGP with more edges and a higher aspect ratio. As a result, the friction between WGP and the cementitious mixture is increased, reducing the workability of the concrete.

To sum up, it could be noted that the differences in particle size, texture, and shape of WGP could lead to opposite effects on the workability of the WGP–cement composites. Smaller and round-shaped WGP would promote the workability, while larger and sharper WGP would reduce the workability.

### 3.2. Setting Time and Hydration

Chandra et al. [57] reported that using up to 30 wt% WGP to replace fine aggregates increased both the initial and final setting time of the cementitious composites. Maglad et al. [52] also found that, after replacing natural sand with 30 wt% WGP, the final setting time increased. They pointed out that, due to the low water adsorption and poor binding between WGP and the fresh cementitious mixture, the presence of WGP slowed the cement hydration, prolonging the setting time of the WGP–cement composites, being in good agreement with other studies [58,59]. When using WGP as a cement replacement, Kamali et al. [37] observed that for up to 20 wt% WGP replacing cement, there were no obvious differences in both initial and final setting times.

Several studies [37,38] found that the presence of WGP as SCMs reduced the heat of the cement hydration. Tiwari et al. [60] pointed out that due to a low pozzolanic reactivity and the cement dilution effect of early age, the setting time of the concrete was increased with the content increase in WGP. Meanwhile, a small content of phosphorus and zinc in WGP acted as retarders in the cement hydration [61]. However, if the particle size of WGP is smaller than 41 µm, there were fewer negative effects on the setting time of the cementitious composites, which may be attributed to the nucleation effects. In general, lower hydration heat would be beneficial for reducing cracks due to excessive temperature during the pouring process of large-scale concrete structures. However, Poutos et al. [62] observed that while using green waste glass powder containing a high amount of Cr_2_O_3_ to replace river sand, the heat generated during cement hydration increased due to the low heat adsorption and pozzolanic reaction of WGP, having a good agreement with Kamali et al. [37].

## 4. Influence on Mechanical Properties

### 4.1. Compressive Strength

Table 3 summarises the potential effects of WGP on the mechanical properties of the WGP–cement composites, highlighting significant differences depending on how WGP are used in cementitious composites. For cement replacement by WGP, Omran & Hamou [63] replaced cement with 20 wt% WGP and found that WGP led to a 15.6% compressive strength increment when compared to WGP-free samples. However, after replacing cement by 10–30 wt%. Similarly, Taha & Nounu [64] reported that a 16% strength reduction was found in samples with 20 wt% WGP (cement replacement) when compared to the reference group, being consistent with other studies [48,65]. He et al. [66] observed that although 10–20 wt% cement replacement by WGP led to a slight compressive strength reduction at 7 days and 28 days, the 90-day compressive strengths of these two groups were increased due to pozzolanic reactions at later ages. Several studies [17,66] pointed out that in the long term, the pozzolanic reactivity of WGP was activated, consuming calcium hydroxide generated from cement hydration to form more C-S-H gel, leading to a denser cementitious microstructure.

For WGP to replace fine aggregates, Chen et al. [71] observed strength increments of 17% and 43% in 28-day and 365-day compressive strength, respectively, in concrete with 40 wt% GWP as fine aggregates. Ismail and AL-Hashmi [69] explained that in the early curing age, the smooth texture of WGP had relatively weak adhesion with the cementitious matrix, reducing the early-age strength development. Another reason for the lower strength development was attributed to the low pozzolanic reactivity of WGP, promoting only filler effects [69]. Furthermore, in geopolymer concrete, the 3-day and 28-day compressive strengths were 55 MPa and 78 MPa, respectively, showing no obvious sign of alkali-silica reactions [72].

### 4.2. Tensile and Flexural Strength

Several studies [10,48,55] reported that the tensile and flexural strengths were reduced due to WGP addition replacing cement or fine aggregate. Limbachiya [70] found that when only a small amount of WGP (<10%) was used to replace fine aggregate, there was no obvious impact on the flexural strength development of WGP–cement composites. However, the author reported that when the WGP content was higher than 20 wt%, the flexural strength gradually reduced, having a good agreement with [73,74]. Ismail and AL-Hashmi [69] found that, if fine aggregates were replaced by 10–20 wt% WGP in the WGP–cement composites, the flexural strength was increased by 3.6–11%, and similar results were found for tensile strength.

Several studies [63,75] observed that using WGP to replace 5 to 25% of cement, the flexural strength of the cementitious composites at a later age was improved by 21 to 49%. Based on the aforementioned results, there is no formal agreement on the potential effects and optimal dosage of WGP on the tensile and flexural strengths of concrete. However, the differences are mainly related to the source, particle size, and type of WGP.

### 4.3. Elastic Modulus

Ali and Al-Tersawy [48] reported that the elastic modulus of concrete with WGP replacing fine aggregates gradually reduced when the WGP content was increased. Limbachiya [70] revealed that there was no obvious difference in elastic modulus between concrete samples with and without WGP. However, He et al. [66] found that when compared to reference concrete, the 28-day elastic modulus of cementitious composites with 10 wt% WGP slightly reduced by 8.6% at an early age. However, at a later age (90 days), the presence of WGP increased the elastic modulus of the concrete specimens by 2.785–4.17%, and 20 wt% WGP as a cement replacement was the optimal dosage to improve the elastic modulus of WGP–cement composites [66]. This is primarily attributed to the pozzolanic reactivity of WGP at a later age, leading to an increase in both strength and elastic modulus. Other studies [63,76] also made a similar conclusion that WGP increased the elastic modulus of WGP concrete, especially at long term (e.g., 90 days).

### 4.4. Dynamic Mechanical Properties

**Figure 3:**
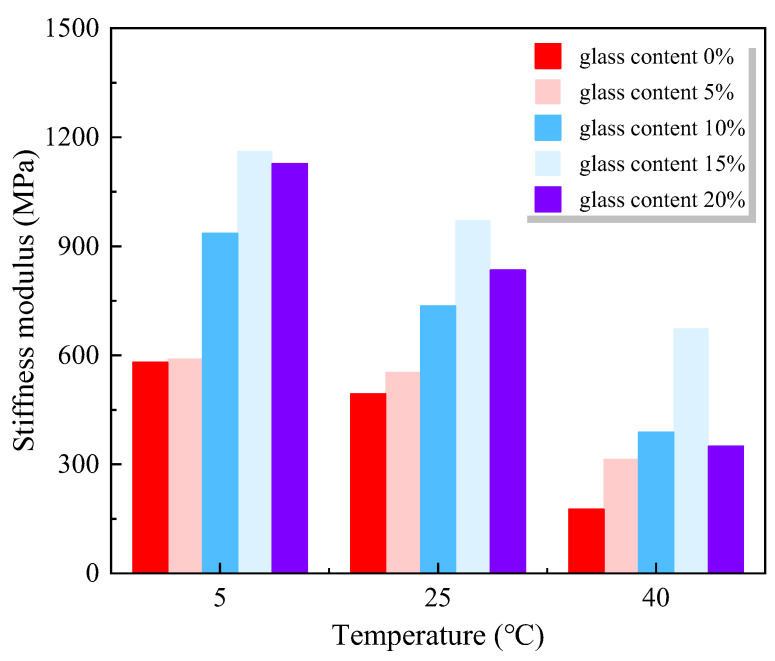
Potential effects of WGP on stiffness of asphalt concrete [78].

Liu et al. [77] investigated the thermodynamic properties of mortar with WGP as a fine aggregate replacement subjected to different vibration frequencies and temperatures. They found that the presence of WGP led to a reduction of 36.5 to 68.5% in the storage modulus of WGP–cement mortar, and the vibration frequency had only a limited influence on the storage modulus. Meanwhile, the loss tangent of the WGP–cement mortar was reduced when the dosage of WGP increased. Figure 3 depicts the potential effects of WGP on the stiffness of asphalt concrete [78]. It could be noted that samples with 15 wt% WGP achieved the best stiffness modulus. Furthermore, Arabani [78] established a stiffness model (Equation (1)) for asphalt concrete with WGP based on WGP dosage and temperature.

(1) S=596+13.1α−10.0T

where *S* refers to the stiffness modules of the asphalt concrete (MPa); α means the dosage of WGP (%), and *T* refers to the temperature (°C).

Similarly, Shafabakhsh and Sajed [79] also observed that using WGP as fine aggregates to partially replace sand increased the stiffness module of asphalt concrete. It is attributed to the sharp edges of WGP, promoting both internal friction and an interlocking effect between WGP and the asphalt matrix.

**Figure 4:**
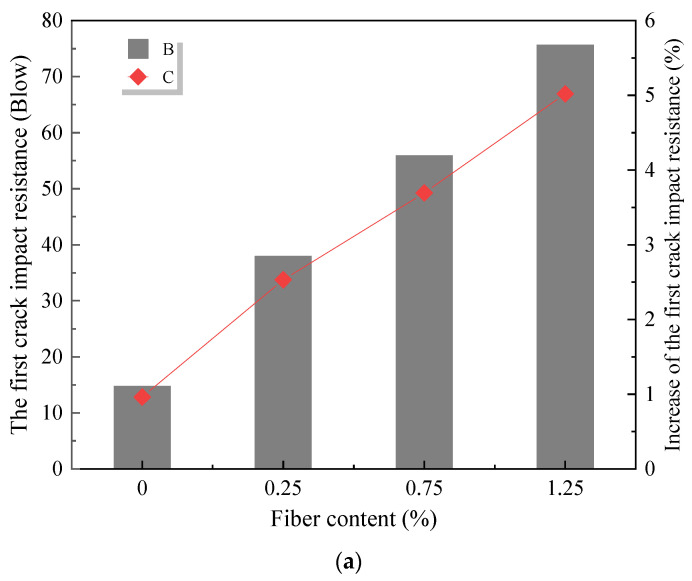

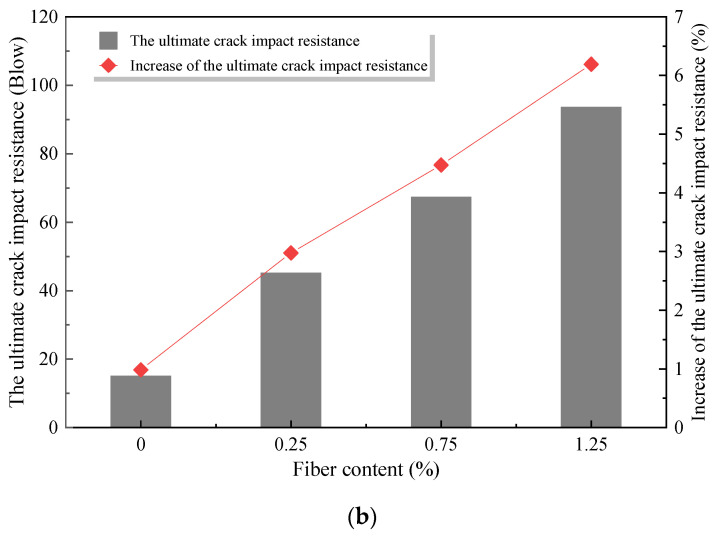
The effect of glass fibre on the impact resistance [78]. (**a**) First crack impact resistance; (**b**) Ultimate crack impact resistance.

Aly et al. [80] analysed the potential effects of WGP as a cement replacement on the impact resistance of mortar. They found that, when compared to the reference group, the impact strength was increased by 39% and 75% for samples with 20 wt% and 40 wt% WGP, respectively. Mastali et al. [81] found that the initial crack impact resistance (Figure 4a) and ultimate crack impact resistance (Figure 4b) were improved by 2.53 to 5.06 times and 2.94 to 6.14 times, respectively, after adding glass fibre.

### 4.5. Discussion Regarding the Effects of WGP on Mechanical Properties

Based on the aforementioned literature, it appears that the mechanism governing the mechanical properties of the WGP–cement composites is not fully understood. Overall, using WGP as SCMs seems to have positive effects on the mechanical properties of WGP concrete, and the main mechanisms are as follows:

Filler effect: When the particle size of WGP is smaller than that of cement powder, the filler effect attributes to blocking pores and voids in the cementitious matrix as well as the interfacial transition zone (ITZ) [82], leading to a reduction in the porosity and critical pore size in WGP concrete. MIP test results [66] indicated that the total porosity of concrete was reduced when the cement was replaced by 10 to 20 wt% WGP, especially the porosity of pores being larger than 100 nm. Similarly, Sobolev et al. [83] also reported that WGP reduced the porosity of WGP mortar. However, excessive content of WGP would increase the porosity of the WGP–cement composites [66].

**Figure 5:**
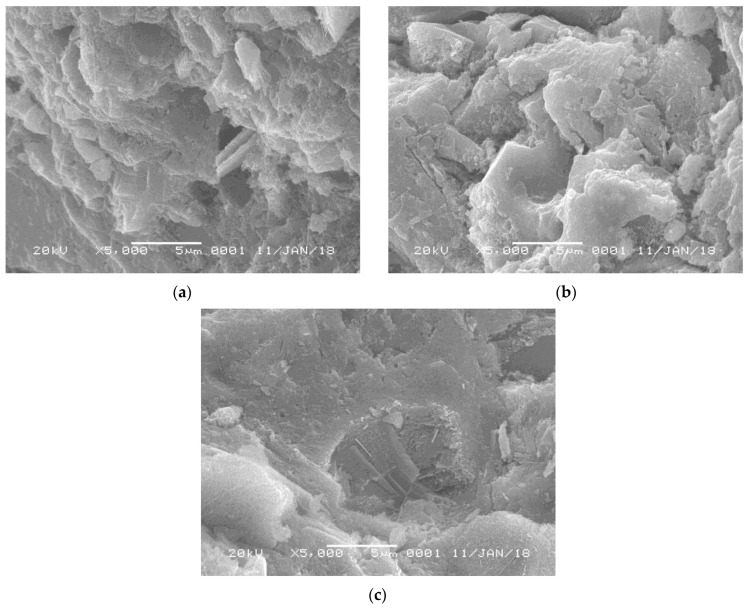
SEM image of 90-day concrete samples. (**a**) Reference group; (**b**) Sample with 10 wt% WGP; (**c**) Samples with 20 wt% WGP.

Pozzolanic reaction [61,71]: Due to the presence of a large amount of amorphous silica in WGP, WGP has the proper potential for pozzolanic reactivity [84]. The pozzolanic reactivity of WGP is related to the particle size [28]. Mirzahosseini and Riding [28] reported that the pozzolanic reactivity of fine WGP (0–25 μm) was higher than that of coarse WGP (25~38 μm or 63~75 μm). Schwarz and Neithalath [85] found that the pozzolanic reactivity of WGP was similar to that of fly ash. A complete pozzolanic reaction could be achieved when the content of WGP is in the range of 30 to 45 wt% [39]. At a later age (90 days), calcium hydroxide (CH) in the cementitious matrix is totally consumed by WGP to form C-S-H gel [86], promoting a denser microstructure [39]. As depicted in Figure 5, when compared to the reference group (Figure 5a), samples with 10 wt% WGP had a more uniform distribution of C-S-H gel and smooth CH crystals, refining the dispersed pores and gel pores (Figure 5b) [66]. He et al. [66] found that when the content of WGP increases, there is a higher degree of pozzolanic reactions, consuming more CH crystal and forming more C-S-H gel [87]. As a result, the hydration products are interconnected and overlapped, leading to a denser microstructure [66]. Meanwhile, WGP promoted pozzolanic reactions, reducing the CH content to form more C-S-H gel, leading to a decrease in the Ca/Si ratio of the cementitious matrix.

**Figure 6:**
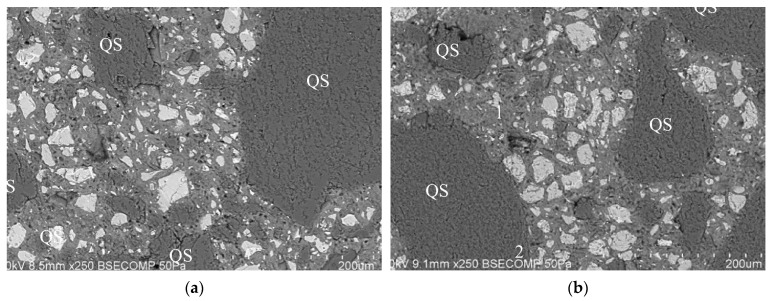
SEM image of ITZ for samples with and without WGP [88]. (**a**) Samples without WGP; (**b**) Samples with WGP.

Proper ITZ bonding: As shown in Figure 6a,b, when using WGP to replace fine aggregates, samples with WGP show no obvious pores or cracks near the WGP particle, indicating a strong ITZ in the WGP–cement matrix [88]. Several studies [50,89] provided similar conclusions. When using WGP to replace cement, an improvement in the bond between the binder and fine aggregates was reported, indicating that WGP could improve the ITZ of the cementitious matrix [39].

In alkali-activated materials: Due to the large content of silica and alkali metal oxides (e.g., Na_2_O), solid silica particles would react with Na_2_O to form sodium silicate gel in geopolymer concrete with WGP [90]. Tho-In et al. [91] observed that the presence of WGP led to an increase in the Si/Al ratio, inducing more formation of aluminosilicate materials and reducing the strength of geopolymer concrete. They also found that samples with 20 wt% WGP had less shrinkage and porosity, having a good agreement with other studies [92,93].

## 5. Influence on Durability

### 5.1. Porosity

**Figure 7:**
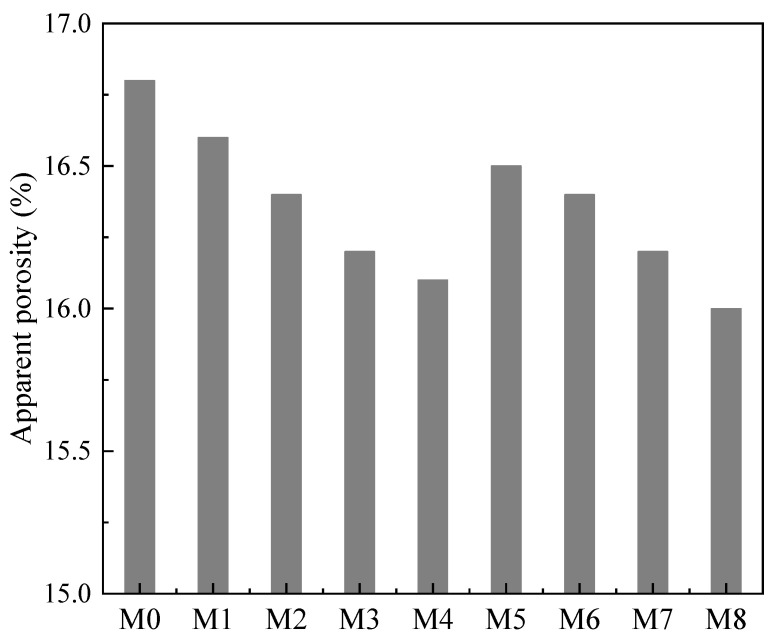
Apparent porosity of samples with WGP addition (Note: M0 means reference group, M1-M4 mean samples with 5 to 20 wt% FA, and M5–M8 refer to samples with 5 to 20 wt% WGP) [94].

WGP used as SCMs can reduce the total porosity of concrete [76]. Yavuz et al. [94] used 5 to 20 wt% WGP to replace cement and found that the apparent porosity of the WGP–cement concrete reduced when the WGP content was increased (Figure 7) based on ASTM C1754. They reported that the apparent porosity was reduced by 5% in samples with 20 wt% WGP. Using Mercury Intrusion Pressure (MIP), You et al. [95] observed that the volume of capillary pores (0.05–0.1 µm) and large pores (>0.1 µm) of samples with WGP reduced when compared to WGP-free samples, due to the formation of additional C-S-H gel refining the pore structure. Vaitkevicius et al. [89] reported that the largest pore was reduced from 700 μm to 70 μm with WGP addition. When the dosage of WGP increases, the void content was firstly reduced and then increased in the cementitious matrix, and the optimal dosage of WGP was 30 wt% [96]. XRD results [89] indicated that the addition of WGP reduced the peaks of CH, C_2_S, and C_3_S, revealing that the cement hydration was increased, reducing the porosity and improving the durability of the WGP concrete. Similarly, Yuan et al. [97] pointed out that using WGP to partially replace cement led to the reduction in the porosity.

### 5.2. Water Absorption

Du and Tan [39] found that concrete water absorption was reduced when using WGP to replace cement, having a good agreement with other studies [57,98]. Patel et al. [99] reported that samples with 20 wt% WGP had a lower water absorption when compared to the WGP-free samples, due to a denser microstructure and lower porosity. Wright et al. [100] completely replaced fine aggregates with WGP and also observed a lower water absorption, being attributed to a lower water permeability due to WGP’s impermeability. Wright et al. [100] found that 1-day water adsorption of samples with WGP was higher than that of samples without WGP, and the 91-day water adsorption was reduced due to WGP addition, being in good agreement with [21,101]. The main reason is that the pozzolanic reactions are activated at later days, refining the cementitious matrix [75,102].

Saberian et al. [103] reported that, with a curing temperature of 50 °C, the water absorption of reference (WGP-free) samples was significantly higher than samples cured at room temperature. By contrast, due to a limited water retention on the surface of WGP, no differences in water absorption of WGP concrete were observed for different curing temperatures [103].

### 5.3. Volume Stability

#### 5.3.1. Shrinkage

**Figure 8:**
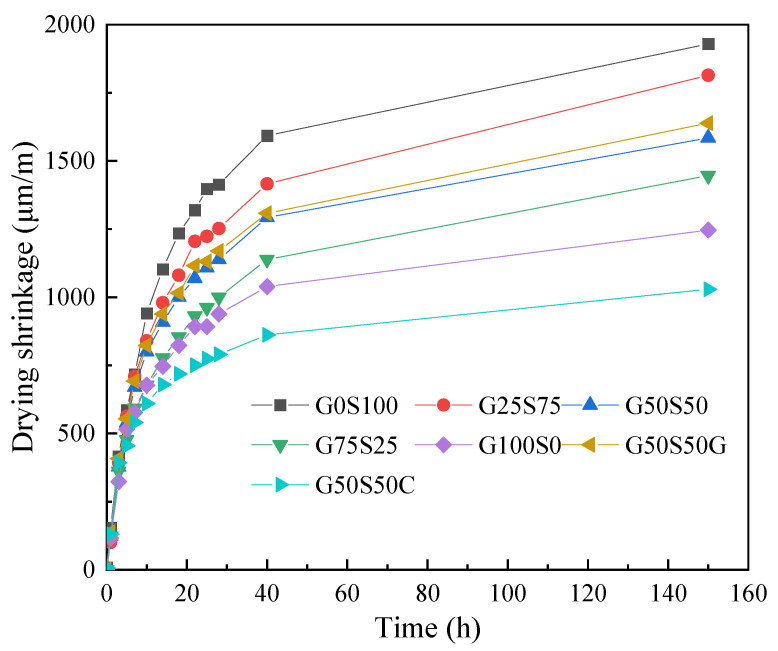
Drying shrinkage of samples with and without WGP [26].

Although numerous studies investigated the shrinkage behaviour of WGP–cement composites, contradictory results are reported when using WGP as an SCM or fine aggregate replacement. Limbachiya [70] reported that using 5 to 10% WGP to replace river sand leads to a reduction in 90-day shrinkage, and excessive dosage of WGP would not have a significant effect on shrinkage. Ling and Poon [104] found that the shrinkage results of samples with and without WGP were very similar up to 28-day exposure, and samples with WGP as fine aggregates showed lower shrinkage. They mentioned that this was attributed to the good cohesion between WGP and the cementitious matrix and the higher elastic modulus of WGP mitigating shrinkage deformation. Similarly, the addition of WGP leads to a reduction in shrinkage of geopolymer concrete [105,106]. Ban et al. [26] found that using WGP as fine aggregates led to the reduction in shrinkage deformations up to 150 days (Figure 8), due to lower water evaporation and a denser microstructure.

**Figure 9:**
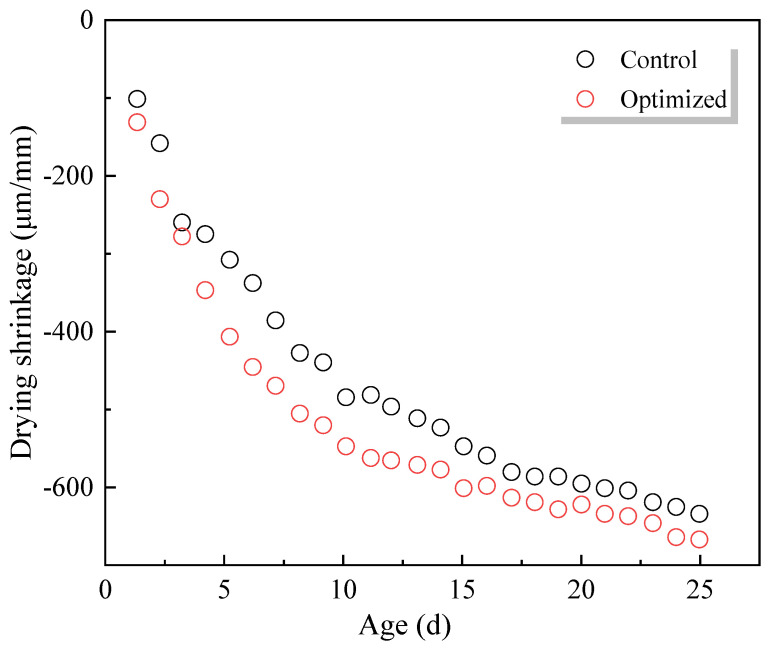
Shrinkage results of cementitious samples [23].

While using WGP to replace cement, Abellan et al. [23] found that the addition of WGP did not lead to a proper mitigation of shrinkage deformation (Figure 9). Likewise, Shayan and Xu [19] observed that when the cement replacement was less than 20 wt%, the shrinkage results of the reference group was similar to those of samples with WGP. When the WGP dosage is higher than 20 wt%, shrinkage gradually increases [19], having a good agreement with the study by Patel [99]. However, Paul et al. [107] reported that using WGP as SCM led to an increase in drying shrinkage when compared to the reference group.

#### 5.3.2. Alkali-Silica Reactions

**Figure 10:**
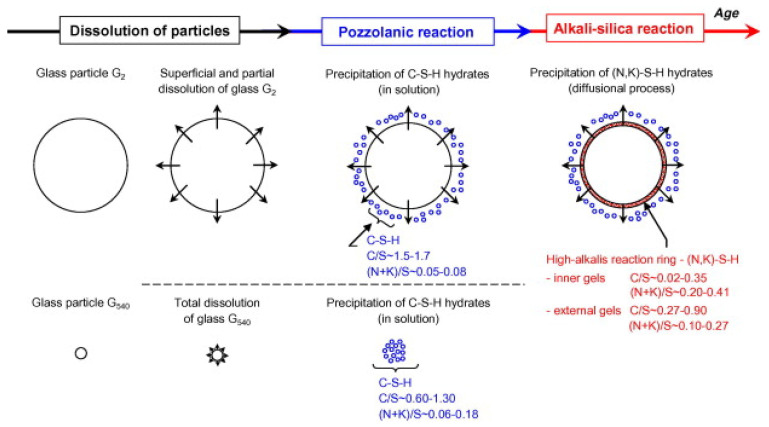
Successive reactions in WGP–cement composite [61].

Due to the large content of amorphous silica in WGP, alkali-silica reactions (ASRs) can be easily triggered, forming expansive siliceous gel, leading to cracks and structural damages in the cementitious composites [108]. Maraghechi et al. [109] noted that the ASRs of WGP were related to residual cracks in the cementitious matrix, and the particle size of WGP affected the reactivity. They reported that no ASRs were found in the microcracks of samples with small-size WGP (<2.5 μm), and ASRs were observed in the microcracks of samples with large-size WGP (>2.5 μm), indicating that samples with larger-size WGP were more prone to ASRs [109]. As shown in Figure 10, Idir et al. [61] supported that small-size WGP would completely dissolve to contribute to pozzolanic reactions and a denser microstructure, and large-size WGP was involved in ASR expansion. Park and Lee [110] reported that the expansion of the cementitious composites increased when the dosage of WGP increased. When compared to samples with green glass, the 14-day expansion of samples with 10 wt% brown glass was increased by 40%, which was attributed to the presence of Cr_2_O_3_ in green glass mitigating the expansion. When the specific surface area of WGP is higher than 4.5 m^2^/kg, the ASR would be mitigated in the WGP–cement composites. Ismail and Al-Hashmi [69] replaced fine aggregates with 10–20 wt% WGP and found that the 14-day ASR expansion of samples with WGP was reduced when compared to the control group. Furthermore, using WGP as SCMs would also reduce the ASR, and the mechanism is that the pozzolanic reactions consume CH and reduce the content of free alkali ions in the pore solution [111], being consistent with the study by Kamali [112].

#### 5.3.3. Creep

There are limited studies on the long-term creep performance of WGP–cement composites. He et al. [66] analysed the influence of WGP as SCMs on concrete compressive creep. They reported that when compared to the control group, the creep of samples with 10 wt%, 20 wt%, and 30 wt% WGP was reduced by 16.1%, 33.6%, and 19.6%, respectively. The main mechanism is that the cementitious microstructure is refined by the filler effect and pozzolanic reactions, leading to a denser microstructure with high-density C-S-H gel [66].

### 5.4. Chemical Resistance

#### 5.4.1. Chloride-Ion Penetration

**Figure 11:**
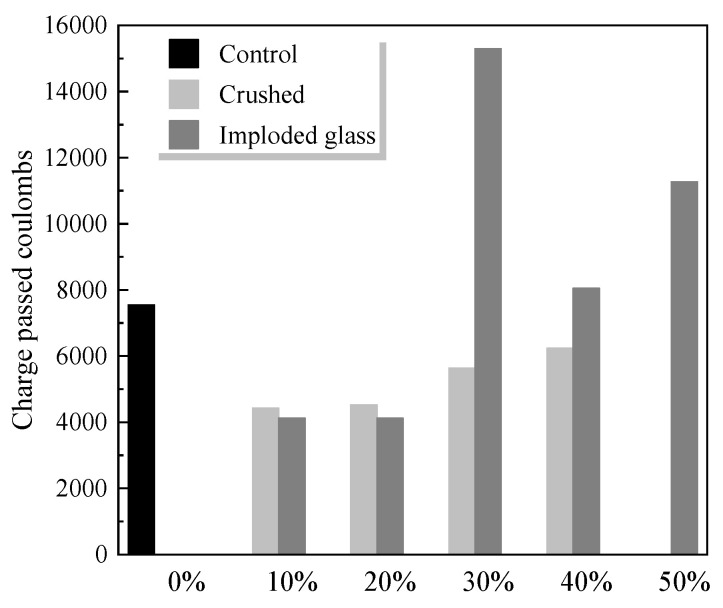
Electrical conductivity results [114].

The pore size distribution, the permeability of aggregates, and the ITZ between aggregates and the cementitious matrix affect the chloride-ion permeability of the cementitious matrix. Table 4 summarises the potential effects of WGP as both SCMs or aggregate replacement on the chloride-ion ingress of WGP–cement composites. Jain et al. [113] found that using WGP as SCM could promote the resistance against chloride-ion ingress of the cementitious matrix. Cassar and Camilleri [114] found that 10 wt% WGP to replace cement led to a 41% reduction in charge passed at 28 days (Figure 11), indicating an improved resistance against chloride-ion ingress. The primary mechanism is that the presence of WGP promotes the pozzolanic reactions, forming more C-S-H gel to reduce the porosity of the cementitious matrix, leading to a reduction in the total charge passing [115]. Matos and Sousa-Coutinho [116] found that, when compared to the reference group, the chloride ion diffusion coefficient of the sample with 10 to 20 wt% WGP as SCM decreased by up to 50%, having a good agreement with Tan and Du [55].

Lee et al. [120] reported that, when using 20 wt% WGP to replace cement, the lowest chloride-ion permeability and total charge passing rate was achieved, being consistent with other studies [15,39,85]. However, Schwarz et al. [21] mentioned that the optimal dosage of WGP to replace cement was 10 wt%, highlighting different performances of WGP–cement composites, which is closely related to the type of WGP, the water-to-binder ratio, and the concrete mix design. Abellan et al. [23] found that using WGP as a cement replacement led to a reduction in chloride permeability at 28 days and 90 days.

For using WGP as a fine aggregate replacement, Liu et al. [117] found that the chloride ion penetration reduced when the dosage of WGP was increased. Liu and Wang [119] also reported that the addition of WGP improved the resistance against chloride-ion penetration of WGP–cement composites. Khan and Sarker [121] suggested that, when using WGP as a fine aggregate, the smooth and water-repellent surface led to a higher impermeability of water and chloride ions. However, Guo et al. [122] observed that using WGP as a fine aggregate did not lead to any significant reduction in the chloride-ion penetration.

Liu and Wang [119] used WGP to completely replace coarse aggregates and found that the chloride-ion permeability was increased by 20%, indicating the different performance of WGP in resisting chloride-ion penetration.

#### 5.4.2. Sulphate Attack

**Figure 12:**
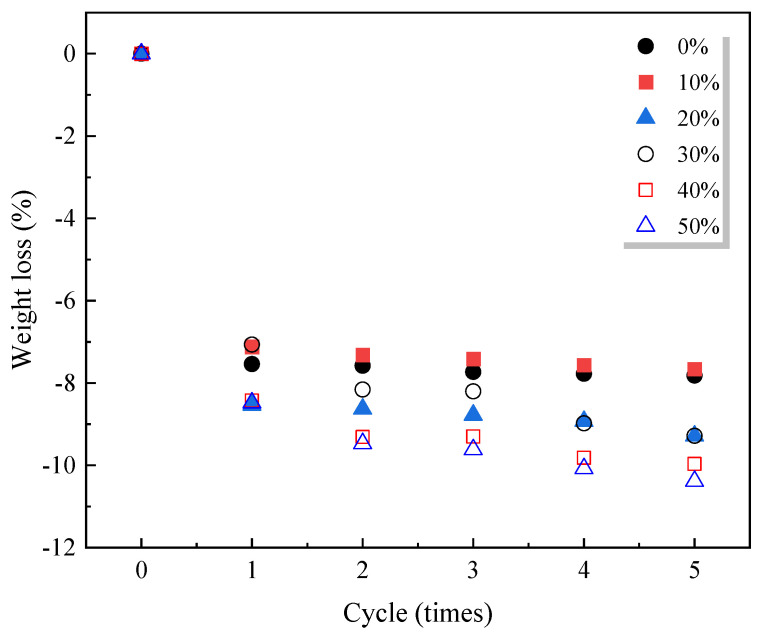
Mass loss of the cementitious samples in sulphate immersion [126].

Harbi et al. [123] reported that using WGP to replace cement by 5 to 25 wt% led to a compressive strength increase up to 180 days, which was attributed to a denser microstructure to resist the sulphate permeability of the cementitious matrix. Özkan and Yüksel [124] observed that, due to the pozzolanic reaction by WGP, the resistance against sulphate attack was increased in WGP–cement composites. However, when the cement replacement was 50 wt%, higher sulphate-ion concentration was found when compared to the reference group [124]. Meanwhile, the presence of WGP negatively affects the resistance to magnesium sulphate due to the transformation of C-S-H gel into magnesium silicate gel, reducing the WGP–cement bond. Matos and Sousa-Coutinho [116] assessed the expansion of sample with and without WGP as SCM immersed in a sulphate solution. They reported that samples with WGP had lower expansion when compared to WGP-free samples. In a similar immersion experiment, Carsan et al. [125] found that WGP as SCMs had the lowest expansion at 1-year immersion when compared to samples with fly ash or GGBFs. However, Wang [126] found that when the cement replacement exceeded 40 wt%, the WGP–cement composites experienced significant mass loss during sulphate immersion (Figure 12), due to both the cement dilution effect and delayed cement hydration.

Wang et al. [127] found that using WGP as a fine aggregate replacement improved the resistance against sodium sulphate ingress, and a better resistance was also observed at a later curing age. The mass loss of samples with 20 to 80 wt% as fine aggregate was reduced by 27 to 61% when compared to WGP-free samples. However, Saccani et al. [128] concluded that using WGP to replace fine aggregate led to no significant improvement in the sulphate resistance of WGP–cement composites.

#### 5.4.3. Acid and Alkali Resistance

Wang [126] reported that using 10 wt% WGP to replace cement led to a higher resistance in sodium hydroxide solution exposure with less mass loss, but the resistance improvement was reduced when the WGP dosage was increased. Similarly, Matos and Sousa-Coutinho [116] found that the optimal dosage to replace cement was 20 wt% WGP in the expansion test with sodium hydroxide immersion, since the resistance was gradually improved when the replacement dosage was up to 20 wt%. Meanwhile, they suggested that WGP–cement composites had better resistance when immersed in calcium hydroxide solution when compared to that in sodium hydroxide, which may be attributed to calcium ions mitigating the dissolution of WGP.

**Figure 13:**
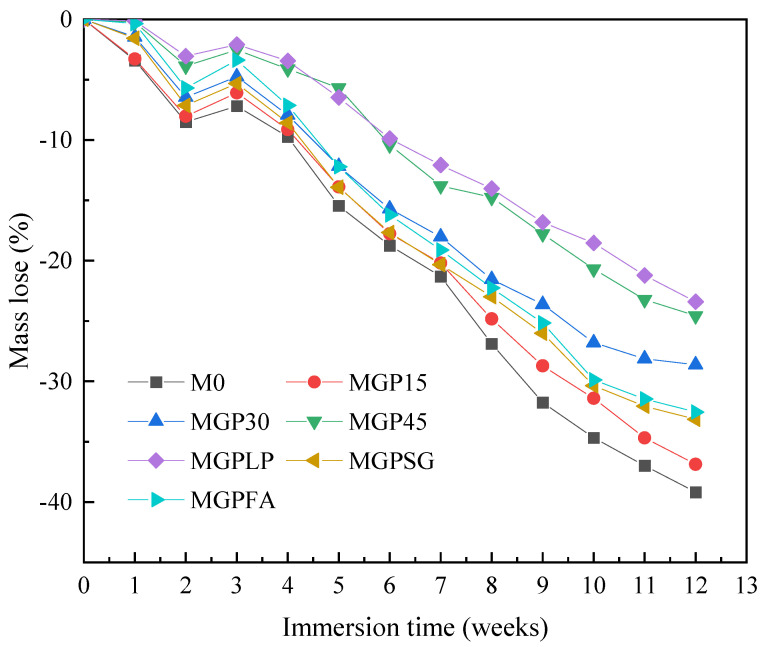
Mass loss of the cementitious samples in 5% sulphuric acid [128]. (Note: M0 refers to control group, MGP15–MGP45 means samples with 15–45 wt% WGP, MGPLP refers to samples with limestone power, MGPSG means samples with GGBFs, and MGPFA means samples with FA).

Siad et al. [129] compared the acid-resisting performance of samples using different SCMs to replace cement, such as WGP, limestone powder, fly ash, and slag. They found that samples with 45 wt% WGP had the best resistance to sulphuric acid after 12 weeks of immersion. As shown in Figure 13, the sulphuric acid resistance is increased when the WGP dosage increases up to 45 wt% [129]. However, Wang et al. [126] pointed out that an excessive dosage of WGP can lower the sulphuric acid resistance due to a less dense microstructure by the cement dilution effect. Lu et al. [41] used WGP as a fine aggregate replacement immersed in 3% sulphuric acid. They found that WGP–cement samples had lower mass loss when compared to WGP-free specimens. In particular, Lu et al. [41] suggested that the reduction in WGP’s particle size could further improve the sulphuric acid resistance of the WGP–cement composites. The main reason is that the formation of additional C-S-H gel densifies the microstructure, and the synergistic effects of polygon-shaped WGP promote the sulphuric acid resistance, having a good agreement with the study by Wang and Huang [130].

#### 5.4.4. Freeze–Thaw Cycle

Yi [131] used WGP as a fine aggregate replacement and found that the presence of WGP led to less freeze–thaw cycle damage in the temperature range of −10 °C to 4 °C, indicating an improved freeze–thaw resistance of WGP–cement composites. Furthermore, Saccani et al. [128] replaced river sand with 20 wt% WGP in geopolymer concrete. They also found that the freeze–thaw resistance was improved in the temperature range of −10 °C to 4 °C.

Abendeh et al. [132] found that when using WGP as a cement replacement (5 to 15 wt%), samples with WGP had lower mass loss after 300 freeze–thaw cycles in the temperature range of −16 °C to 6 °C when compared to WGP-free samples, being consistent with the study by Lee et al. [120]. They reported that the freeze–thaw resistance depended on the porosity and strength of the cementitious composites, rather than the filler effects and pozzolanic reactions by WGP. Other studies [101,133,134] reported that using WGP as SCMs (<20 wt%) increased freeze–thaw resistance.

Mardani-Aghabaglou et al. [135] found that when the dosage of WGP replacing cement increased, the dynamic elastic modulus of WGP–cement composites reduced after the freeze–thaw cycle. Dong et al. [136] reported that the primary reason was that temperature variance led to degradation of the cementitious matrix, leading to a strength loss. However, Kim et al. [137] pointed out that using WGP as a fine aggregate replacement did not lead to any significant differences in freeze–thaw resistance.

Therefore, the presence of WGP as a fine aggregate replacement is beneficial in freeze–thaw resistance, while contradictory results were observed when using WGP as SCM.

#### 5.4.5. Elevated Temperature

Belouadah et al. [138] reported that using WGP as a cement replacement up to 10 wt% led to higher initial and residual compressive strength of WGP–cement composites cured in a chamber with a temperature of 600 °C for 1 h. They explained that the elevated temperature accelerated the cement hydration and increased the pozzolanic reactivity of WGP, leading to a lower compressive strength loss. Similarly, Pan et al. [139] found that when the testing temperature was lower than 500 °C for 1.5 h, the strength loss of samples with and without WGP was 15% and 33%, respectively, indicating an improved resistance to elevated temperature of the WGP–cement composites. Meanwhile, Pan et al. [139] added that in curing temperatures of 500–800 °C for 1.5 h, the strength of the WGP sample was 21% higher than that of the WGP-free samples, having a good agreement with the study by Lu et al. [41]. Ali et al. [140] mentioned that when compared to samples with silica fume, samples with WGP had higher strength after being exposed to elevated temperatures, showing less matrix degradation.

Yang et al. [141] found that when using WGP as fine aggregates, samples with small-size WGP had better resistance to elevated temperatures, being consistent with the study by [142]. The primary reason is that under high temperatures (>500 °C), the melted surface of the WGP particle could fill cracks and voids to refine the cementitious matrix, reducing the high-temperature damage in the ITZ between WGP and the cementitious matrix [143]. Zhang et al. [144] also reported that the presence of WGP reduced the strength loss of the cementitious samples subjected to elevated temperatures.

## 6. Conclusions and Outlook

This study systematically analyses the potential effects of using WGP to partially replace cement or aggregates on both the mechanical and durability properties of WGP–cement composites, aiming to reduce the carbon footprint and sustainability of concrete structures. The main conclusions are as follows:

1. The differences in WGP’s particle size, texture, and shapes could lead to various effects on the workability and hydration of the WGP–cement composites.
2. 10–20 wt% WGP as SCMs seems to be the optimal dosage to improve the mechanical strength of WGP–cement composites. Moreover, at a later age, WGP could promote more strength improvements, being attributed to activated pozzolanic reactions and filler effects.
3. The addition of WGP as SCMs reduces the porosity and lowers the water adsorption of the WGP–cement composites.
4. When replacing cement by up to 20 wt% WGP, the shrinkage, ASRs, and creep are reduced, being attributed to the formation of more C-S-H gel reducing the porosity.
5. The presence of WGP as SCM promotes the resistance to chloride-ion penetration, sulphate attack, acid and alkali resistance, freeze–thaw damage, and elevated temperature damage.

By considering the aforementioned literature, even though numerous studies were conducted to investigate the potential effects of WGP on the properties of WGP–cement composites, there are still many issues related to the durability of concrete with WGP, since contradictory conclusions are reported. Thus, the perspectives are as follows:

1. The chemical composition of WGP can be very different, leading to different pozzolanic reactivity, concrete durability, and ASR reactivity. Hence, it is necessary to establish a comprehensive WGP classification (composition, processing, mixing, etc.) and link them to specific potential concrete applications.
2. There are contradictory conclusions on the effects of WGP on the durability properties of cementitious composites. As a result, it is suggested that more studies are needed to provide conservative conclusions and guidance on how WGP may affect concrete durability depending on the WGP characteristics.
3. Studies on the usage of WGP in high-strength concrete are limited; further investigations are required.
4. There is a lack of quantitative evaluation of both economic and environmental impacts of WGP. More studies on life cycle analysis are recommended.
5. It is suggested to conduct numerical analyses to establish models allowing to predict the performance of WGP–cement composites.
6. The outlooks for WGP applications are as follows:
7. Due to the intensive use of concrete in structural and infrastructure design, there is a need for improved concrete properties with lower shrinkage and permeability while promoting the structural resilience of the concrete structure.
8. The needs of sustainable concrete design require the usage of low-carbon and green materials to reduce the carbon footprint. There would be an increasing trend in recycling WGP as both a cement and aggregate replacement, considering the reduction in cost and resources in the WGP landfill process.

## Figures and Tables

**Table 1:** Chemical compositions of WG.

SiO_2_	Na_2_O	CaO	Al_2_O_3_	K_2_O	MgO	LOI	Refs.
20.3	0.23	63.8	4.68	0.82	1.98	8.19	Cement [29]
70.65	11.72	10.59	1.58	0.52	0.68	4.26	Waste glass fume [29]
59.7	14.7	18.2	1.31	0.35	3.59	2.15	Waste window glass [25]
74.11	12.4	10.01	0.01	0.25	2.6	0.62	Waste glass bottle [30]
67.7	14.75	11.7	2.4	0.81	0.49	2.15	Waste glass bottle [31]
72.75	12.45	7.62	1.48	0.43	3.1	2.17	Waste glass bottle [32]
69.3	14.7	9.1	2.7	0.8	1.5	1.9	Waste brown glass [22]
68.4	13.5	10.8	0.9	0.3	1.8	4.3	Waste soda-lime glass [33]
54	8.7	0.85	2.5	8.5	0.4	25.05	Waste tube glass [26]
72.68	10.6	11.84	1.57	0.5	1.69	1.12	Waste glass [3]
73.6	12.6	8.04	1.44	0.34	2.78	1.2	Waste green glass [24]
72.23	12.9	9.58	0.13	0.32	3.43	1.41	Commercial glass powder [34]
72.59	11.61	9.42	1.41	0.72	1.55	2.7	Commercial glass powder [23]
72.4	12.25	10.32	1.85	0.65	2.09	0.44	Commercial glass powder [35]
87.46	5.8	2.68	0.25	0.1	2.46	1.25	Commercial glass powder [36]

**Table 2:** Effects of WGP on the fresh properties.

Type	Replacement (wt%)	Effects	Standard	Ref.
Supplementary cementitious materials (SCMs)	20	Flow reduced by 29.0%	BS EN1015 [44]	Lu et al. [13]
	20–30	7.1–14.3% flow reduction	Not mentioned	Shayan & Xu [19]
	5–30	Flow increased by 20–100%	ASTM C 1437 [45]	Schwarz et al. [43]
Fine aggregates	25~100	Increased by 8.3–26.9%	BS EN1015 [44]	Lu et al. [46]
	10–50	Slump increased by 4.7–37.5%	ESS 2421/2005 [47]	Ali & Al-Tersawy [48]
	18–24	Reduced the workability	IS: 1199-1959 [49]	Bisht & Ramana [50]
	30–70	Workability reduced by 19.6–44.3%	KS F 2402 [51]	Park et al. [14]

**Table 3:** Effects of WGP as fine aggregates and cement replacement on the mechanical properties of the cementitious composites.

	Type	Replacement Percentage (%)	Age (Days)	Testing Programme	Strength Increment	Ref.
Mortar	Fine aggregates	20–100	28	Compressive strength	11.5–16.4%	Penacho et al. [67]
				Flexural strength	5–22.7%	
		10–40	28	Compressive strength	−(4–75%)	Li et al. [68]
		20	28	Compressive strength	15.6%	Omran and Tagnit-Hamou [63]
				Tensile strength	22.5%	
				Flexural strength	13.8%	
		10–25	28	Compressive strength	−(5–13%)	SadiqulIslam et al. [15]
Concrete	Fine aggregates	10–20	28	Compressive strength	−8.4~4.3%	Ismail and AL-Hashmi. [69]
				Flexural strength	3.57–11.2%	
		20–50	28	Compressive strength	2–30%	Limbachiya et al. [70]
				Flexural strength	40–150%	
		50	28	Compressive strength	−(1.3–2.6%)	Taha and Nounu. [64]
		10–40	28	Compressive strength	10~60%	Chen et al. [71]

**Table 4:** Chloride-ion ingress of WGP concrete.

Usage	Percentage (%)	Age (Days)	Resistance Improvement	Testing Programme	Refs.
SCMs	10	28	41%	Rapid chloride penetration test (RCPT)	[114]
	30	380	53%	RCPT	[19]
	10	90	14%	RCPT	[21]
	15	56	91%	RCPT, rapid chloride migration (RCM)	[39]
Fine aggregates	60	45	17.5%	RCPT	[117]
	50	28	7%	RCPT	[55]
	20	28	61%	RCPT	[118]
Coarse aggregates	100	210	−20%	Chloride ion diffusion	[119]

## Data Availability

The original contributions presented in this study are included in the article. Further inquiries can be directed to the corresponding authors.
